# Cadherin 8 regulates proliferation of cortical interneuron progenitors

**DOI:** 10.1007/s00429-018-1772-4

**Published:** 2018-10-12

**Authors:** Fani Memi, Abigail C. Killen, Melissa Barber, John G. Parnavelas, William D. Andrews

**Affiliations:** Department of Cell and Developmental Biology, University College London, Gower Street, London, WC1E 6BT UK

**Keywords:** Cell migration, Cadherin, Interneurons, Proliferation

## Abstract

Cortical interneurons are born in the ventral forebrain and migrate tangentially in two streams at the levels of the intermediate zone (IZ) and the pre-plate/marginal zone to the developing cortex where they switch to radial migration before settling in their final positions in the cortical plate. In a previous attempt to identify the molecules that regulate stream specification, we performed transcriptomic analysis of GFP-labelled interneurons taken from the two migratory streams during corticogenesis. A number of cadherins were found to be expressed differentially, with Cadherin-8 (Cdh8) selectively present in the IZ stream. We verified this expression pattern at the mRNA and protein levels on tissue sections and found approximately half of the interneurons of the IZ expressed Cdh8. Furthermore, this cadherin was also detected in the germinal zones of the subpallium, suggesting that it might be involved not only in the migration of interneurons but also in their generation. Quantitative analysis of cortical interneurons in animals lacking the cadherin at E18.5 revealed a significant increase in their numbers. Subsequent functional in vitro experiments showed that blocking Cdh8 function led to increased cell proliferation, with the opposite results observed with over-expression, supporting its role in interneuron generation.

## Introduction

Interneurons constitute a morphologically, neurochemically and functionally diverse group of cortical neurons that are essential modulators of neuronal activity in the cerebral cortex. Abundant evidence indicates that alterations in the number, distribution and function of these GABA-releasing inhibitory neurons in humans may lead to neurological and psychiatric disorders, including some of developmental origin (Benes and Berretta 2001; Cossart et al. 2005; Gant et al. 2009). Thus, a great deal of effort has been devoted to understanding the mechanisms that control their development.

Whilst many molecules have been identified as regulators of interneuron migration (Faux et al. 2012; Marin 2013; Guo and Anton 2014), little is known about the factors that determine their choice of tangential migratory stream as they enter the cortex. Previously, we reasoned that molecular/genetic differences between interneurons underlie their choice of one of the two early tangential pathways. To identify genes involved in migratory stream specification, we previously compared the gene expression profiles of cells in the pre-plate (PPL) zone with those of cells migrating through the IZ during early corticogenesis (E13.5). Our analysis identified several cadherin family members that showed differential expression, including Cdh8, which was present only in the IZ at this stage (Antypa et al. 2011), a finding recently supported by another study (Pensold and Zimmer 2018).

Cdh8 is a classical type II cadherin that can bind β-catenin (Kido et al. 1998). Whilst many type-II cadherins are expressed rather ubiquitously during CNS development, there are a few amongst their number that exhibit restricted expression patterns that are spatially and temporally regulated (Lefkovics et al. 2012). Most studies examined Cdh8 expression perinatally and postnatally, when it is expressed strongly in certain layers of the cortex, hippocampus and striatum (Korematsu and Redies 1997a, b; Korematsu et al. 1998b; Medina et al. 2004; Lefkovics et al. 2012). Differential expression of Cdh8 within the striatum has been suggested to aid segregation and aggregation of cell types (Korematsu et al. 1998a). Cdh8 has also been found to have a role in the migration and growth of other neuronal cell types (Garel et al. 2000; Taniguchi et al. 2006; Bekirov et al. 2008), and to have a function in the establishment and remodelling of synapses and in coupling between neurons (Suzuki et al. 2007; Bekirov et al. 2008; Huntley et al. 2012). On the basis of these findings, we postulated that Cdh8 could have many roles in interneuron development.

Here we carried out a detailed expression analysis of Cdh8 throughout the whole period of corticogenesis and analysed the interneuron phenotype in Cdh8 knockout animals. We observed differences in the number of interneurons in these mice and assessed the roles of Cdh8 in cell migration, apoptosis and proliferation. Our findings identified a novel function for this cadherin in cortical interneuron development.

## Materials and methods

### Animals

All experimental procedures were performed in accordance with the UK Animals (Scientific Procedures) Act 1986 and institutional guidelines. Wild-type animals were C57/bl6J mice obtained from Charles River Ltd. *Cdh8*^−/−^ knockout (KO) mice and *GAD67-GFP*^*neo*/−^ mice were generated as described previously (Tamamaki et al. 2003; Suzuki et al. 2007). The day the vaginal plug was found was considered as embryonic day (E) 0.5. Animals of both sexes were used in our experiments.

### Cell lines

COS-7 cells (American Type Culture Collection) and GN11 cells were grown as monolayers at 37 °C in a humidified CO_2_ incubator in DMEM (ThermoFisher Scientific) and supplemented with 10% foetal bovine serum (FBS) (ThermoFisher Scientific) and 100 U/ml penicillin/streptomycin (Gibco). Subconfluent cells were harvested by trypsinization and cultured in 10 cm^2^ dishes. Media was changed to low-serum Optimem (Gibco) 24 h prior to collection of conditioned media for Western blot and chemotaxis migration assays. Cells within six passages were used in all experiments.

### In situ hybridization

For in situ hybridization and immunohistochemistry, embryonic brains were dissected in PBS and fixed in 4% paraformaldehyde (PFA), made in phosphate buffered saline (PBS), for 4–8 h at room temperature (RT). Following fixation, embryonic brains were cryoprotected in 30% sucrose in diethyl pyrocarbonate (DEPC) treated PBS, embedded and frozen in a mixture of 15% sucrose/50% Tissue-Tek OCT (Sakura Finetek), and sectioned in the coronal plane at 20 µm using a Cryostat (Bright Instruments). Sections were dried at RT for 2 h before overnight incubation at 65 °C in hybridization buffer [1 × DEPC treated ‘Salts’ (200 mM NaCl, 5 mM EDTA, 10 mM Tris pH 7.5, 5 mM NaH_2_PO_4_.2H_2_O, 5 mM Na_2_HPO4; Merck); 50% deionized formamide (Ambion); 0.1 mg/ml RNAse-free yeast tRNA (ThermoFisher Scientific); 1 × Denhardts (RNase/DNase free; ThermoFisher Scientific); 10% dextran-sulphate (Merck)] containing 100–500 ng/ml DIG labelled-RNA probes. Probes were generated by linearization of plasmids with appropriate enzymes and reverse transcription polymerases to obtain antisense probes. *Cdh8* and *Lhx6* probes were kindly provided by Prof. Gregor Eichele (Max Planck Institute for Biophysical Chemistry, Göttingen, Germany). Following hybridization, sections were washed 3 times in 50% formamide 1XSSC (Ambion) and 0.1% Tween-20 (Merck) at 65 °C and two times at RT in 1XMABT (20 mM Maleic acid, 30 mM NaCl, 0.1% Tween-20; Merck) before incubating in blocking solution [2% blocking reagent (Roche), 10% normal goat serum (Vector Laboratories) in MABT] followed by overnight incubation in alkaline phosphatase conjugated anti-DIG antibody (1:1500; Roche). Nitro blue tetrazolium chloride/5-bromo-4-chloro-3-indolyl phosphate (Roche) diluted 1:1000 in MABT with 5% polyvinyl alcohol (VWR International Ltd) was used for the colorimetric detection for 6 h. Fast Red (Roche) was used for fluorescent colour detection of probes by incubation in 100 mM Tris (pH 8.0) and 400 mM NaCl containing Fast Red at 37 °C for approximately 2 h. Fluorescent in situ hybridization was followed by immunohistochemical detection of GFP as described below. Sections were mounted with Glycergel Mounting Medium (Dako).

### Immunohistochemistry

Embryonic brain sections were washed in PBS, blocked in a solution of 5% normal goat serum (Merck) (v/v) containing 0.1% Triton X-100 (v/v) (Merck) in PBS at RT for 2 h. They were subsequently incubated in primary antibodies at RT for 2 h and then at 4 °C overnight. The following antibodies were used: mouse monoclonal Nestin (1:100, DSHB) and 5-Bromodeoxyuridine (BrdU; 1:1000, Progen), Cad8-1 (1:100, DSHB), chicken polyclonal raised against GFP (1:500, Aves Laboratories), rabbit polyclonal raised against calbindin (CB-28; 1:3000, Swant), cleaved caspase-3 (CC3; 1:250, Cell Signaling Technology), Ki67 (1:1000, Cell Signaling), Tbr2 (1:500, Abcam) or phosphohistone H-3 (PH-3; 1:1000, Millipore). For blood vessel staining, sections were incubated with biotinylated Griffonia (Bandeiraea) Simplicifolia lectin I (Isolectin B4) (1:200, Vector) followed by fluorescent Strepatividin-405 (1:200, Vector Laboratories).

### Interneuron counts

In Cdh8 knockout sections at E18.5, 300 µm was measured along the ventricular surface of the cortex next to the cortico-striatal junction. A rectangle was then drawn to incorporate the entire thickness of the cortex within the 300 µm, and the number of stained cells in that area was counted. We quantified the number of interneurons in each layer of the cortex as well as the total number.

### Plasmids and RNAi constructs

For over-expression experiments, we first generated mouse cDNA from E13.5 mouse forebrain mRNA obtained using RNAeasy kit (Qiagen), and Superscript III cDNA synthesis kit (Invitrogen). Mouse *Cdh8* cDNA was produced by PCR amplified using *Pfu* polymerase (Promega) (Forward (*Nhe*I), GCTAGCGATGCCAGAAAGGCTTGCTGAGACGC; Reverse (*Kpn*I), CCACTTTCACTGTTTCTTTGACCATGGTTC), digested with *Nhe*I and *Hin*dIII and subcloned into the pCDNA3.1 (–) expression vector (Promega). For RNAi experiments, we designed four different oligonucleotides; targeting specific regions of mouse *Cdh8* cDNA (GenBank accession number NM_007667.3), S1 specifically recognizes nucleotides 903–921, GCTGGCACAATCTTTCAAA; S2, nucleotides 1554–1569, GCACTATTCGAAATCA; S3, nucleotides 1917–1935, GCAGATGATGGGAAGATAA; S4, nucleotides 2078–2097, GCGCATCCGAATATGAGGCAT; S1m, nucleotides GCTGGCA**GT**ATCT**A**TCAAA, nucleotides highlighted in bold and underlined were altered from sequence S1.

Annealed oligonucleotides were cloned in the GeneClip™ U1 Hairpin-hMGFP vector according to the manufacturer’s instructions (Promega). As controls, we used short interfering RNAs (siRNAs) targeting the same regions, but containing three-point mutations and, thus, not affecting the stability of *Cdh8* mRNA. The efficiency of the different siRNAs in targeting *Cdh8* mRNA was determined by co-transfecting mouse *Cdh8* cDNA and the different siRNAs at a ratio 1:3, using Lipofectamine 2000 (Invitrogen), into COS-7 cells. After 48 h, mRNA and protein were harvested and the level of knockdown determined.

### qPCR

cDNA from transfected and MGE cells was analysed by qPCR. The qPCR reaction was performed with SYBR Green reagent (Sigma) on a CFX96™ Real-Time PCR Detector system (Bio-Rad) in accordance with the manufacturer’s instructions. Primers for quantitative realtime PCR (QPCR) were designed by Sigma-Genosys and were as follows: β*-Actin* (forward, GGCTGTATTCCCCTCCATCG; reverse, CCAGTTGGTAACAATGCCATGT);

*Cdh8* (forward, TGCATGAGGCAGATAATGACCC; reverse, TCTGGTCTGAGTCTGATGTGG); *Ccd1*(forward GCGTACCCTGACACCAATCTC; reverse CTCCTCTTCGCACTT-CTGTC); *Gad1* (forward CACAGGTCACCCTCGATTTTT; reverse ACCATCCAACGAT-CTCTCTCATC); *Gapdh* (forward AGGGCATCTTGGGCTACAC; reverse CATACCAGG-AAATGACGTTGA); *Ki67* (forward ATCATTGACCGCTCCTTTAGGT; reverse GCTCGCCTTGATGGTTCCT); *Sox2* (forward CGGCACAGATGCAACCGAT; reverse CCGTTCATGTAGGTCTGCG); GAPDH and β-actin were used for endogenous reference gene controls. Each primer set amplified a single PCR product of predicted size as determined by melt-curve analysis following PCR and by agarose gel electrophoresis and had approximately equal amplification efficiencies when validated with a serial dilution of representative cDNA. Each qPCR was performed in triplicate, and relative quantification was determined according to the DDc(t) method (Livak and Schmittgen 2001; Faux et al. 2010).

### Expression of Cdh8

COS-7 cells were lysed in lysis buffer (50 mM Tris–HCl pH 7.4, 150 mM NaCl, 1% Nonidet P40). The lysate was incubated at 4 °C for 30 min and centrifuged for 5 min. Cell lysates were processed for conventional SDS-PAGE and membrane transfer. To assess the protein levels of Cdh8 and Gapdh, membranes were incubated with the following polyclonal antibodies: goat anti-Cdh8 (1:1000; Santa Cruz), and Gapdh (1:500; Sigma-Aldrich), in 5% BSA-TBST, washed several times with TBST, and incubated with a horseradish peroxidase-conjugated secondary antibody (1:5000; Vector Laboratories). After intensive washing, the proteins were visualized with ECL detection reagent (GE Healthcare).

### Dissociated MGE cell cultures

Dissociated cell cultures were prepared from embryonic rodents as described previously (Cavanagh et al. 1997). Briefly, MGEs were dissected out in cold artificial CSF (ACSF) under a stereo microscope. They were incubated in trypsinization medium [0.05% trypsin (Merck) with 100 µg/ml DNaseI (Roche) in neurobasal medium (ThermoFisher Scientific)] at 37 °C for 15 min. Trypsinization was quenched with neutralization medium (10% of FBS, ThermoFisher Scientific, in neurobasal medium) at 37 °C for 5 min. MGEs were then triturated by pipetting until no cellular aggregates were visible. The homogenous cell suspensions were subsequently pelleted by centrifugation at 1000×*g* for 3 min. Cells were resuspended in dissociated culture medium (DM) [DMEM/F12 media containing 10% of FBS and B27 supplement (ThermoFisher Scientific), 100 µg/ml penicillin/streptomycin (ThermoFisher Scientific), and 2 mM l-glutamine (ThermoFisher Scientific)] and 1,000,000 cells were seeded on to 13 mm coverslips coated previously with 10 µg/ml poly-l-lysine and 10 µg/ml laminin and incubated in a humidified incubator at 37 °C. The culture medium was changed the next day.

### Chemotaxis assays

Chemotaxis assays were performed using a 48-well Boyden’s chamber (NeuroProbe) as described previously (Killen et al. 2017). Briefly, 27 µl of DM containing 10% FBS were placed into the lower compartment of the chamber. Dissociated MGE or GN11 cells were re-suspended in serum-free medium (10^5^ cells/50 µl) and placed in wells of the upper compartment of the chamber. These were separated from the lower chamber by a polycarbonate porous membrane (8 µm pores), pre-coated with gelatin (0.2 mg/ml) for GN11 cells and laminin (10 µg/ml) for MGE cells. The chamber was kept in an incubator at 37 °C for 4 h (GN11 cells) or overnight (MGE cells). After incubation, the migrated cells that adhered to the underside of the membrane were fixed and stained using the Diff-Quick kit (Reagena). For quantitative analysis, the membranes were observed using an Olympus light microscope with a 20 × objective adapted with a 500 × 500 µm grid. Four random fields of stained cells were counted for each well, and the mean number of migrating cells per square millimeter for each experimental condition was estimated.

### Proliferation/apoptosis experiments

For apoptosis experiments, 24 h following transfection of cells with over-expression and knockdown constructs, media was changed to DMEM (ThermoFisher Scientific) without FBS. Next day cells were washed, fixed with 4% PFA, immunostained for PH3 and Nestin to identify progenitors, and EGFP to identify transfected cells. For analysis of apoptosis in these cultures, CC3 and calbindin immunohistochemistry was carried out instead of PH3. The percentage of transfected cells immunoreactive for Nestin and PH3, or calbindin and CC3, was counted using a 40 × objective lens in nine fields of view for each sample. At least three samples were evaluated for each time point and treatment. For over-expression constructs, the control was cells that had been transfected with empty pcDNA3.1 (ThermoFisher Scientific) vector. For knockdown constructs, the controls were cells that had been transfected with mutated siRNA constructs in the same pGeneClip™ hMGFP vector.

### BrdU experiment

BrdU was added to culture medium at a final concentration of 3 µg/ml for 2 h and cells or slices were fixed for 30 or 60 min at 4 °C, respectively, with 4% PFA. They were then washed three times with PBS and treated in 2 N HCl for 30 min. After 3× washes, cells or slices were processed for anti-BrdU immunostaining. Cell cycle length was calculated as the ratio of BrdU^+^Ki67^+^/Ki67^+^ and exit from the cell cycle as the ratio of BrdU^+^Ki67^−^/BrdU^+^ in MGE cell cultures.

### Brain slices

Brains slices were obtained with Vibratome (Leica VT1000s) and cultured as described previously (Andrews et al. 2013). Briefly, medial coronal brain slices, obtained from E13.5 mice were placed onto nitrocellulose filters (0.45 µm; Millipore) in culture medium containing DMEM:F12 (Sigma,-Aldrich), 5% foetal bovine serum, 1× N-2, 1× B-27, 100 µM l-glutamine, 2.4 g/l d-glucose (Sigma-Aldrich), 5 U/ml penicillin, and 5 mg/ml streptomycin in a humidified 5% CO_2_ incubator at 37 °C in the presence of 1 µg/ml human IgG (Merck) or 1 µg/ml Cdh8-Fc (R&D Systems). 24 h later, they were fixed with 4% PFA in 0.1 M phosphate buffer, pH 7.4, at 4 °C for 1 h, before carrying out immunohistochemistry for Ki67 and pH3. Slices were mounted on slides using Mowiol 4–88 and imaged with SP8 confocal microscope (Leica Microsystems). Images were taken at the level of MGE, and Photoshop CS6 was used for cell quantification. A box of 10^4^ µm^2^ surface was drawn at the VZ or SVZ and the number of stained cells in that box was counted.

### Digital image acquisition and processing

Optical and fluorescent images were collected using a Leica Microsystems light microscope (DM5000B), and with the SP8 confocal microscope (Leica Microsystems). Images were reconstructed and digitized with Photoshop CS6 software (Adobe Systems).

### Statistics

Statistical analyses were performed by GraphPad 3 (Graph-Pad Software). All data are reported as mean number and SEM. The statistical significance between group means was tested by one-way ANOVA, followed by Bonferroni’s post hoc test (for multiple comparison tests) or Student’s *t* test for paired comparisons. Significance was set at *p* ≤ 0.05.

## Results

We previously carried out a microarray analysis to identify genes that showed differential expression between the early tangential migratory streams, which may underlie the choice of pathway for migrating cortical interneurons (Antypa et al. 2011). This analysis identified several differentially expressed cadherin molecules, including Cdh8, which appeared to be expressed specifically in the IZ during early corticogenesis (E13.5). Previous studies have given a fairly detailed account of *Cdh8* expression perinatally and, especially, postnatally in the developing rodent brain (Korematsu and Redies 1997a, b; Korematsu et al. 1998b; Medina et al. 2004; Lefkovics et al. 2012), but little is known about its expression in the early-mid developing cortex. We, therefore, undertook a detailed analysis of *Cdh8* expression in the embryonic mouse telencephalon, focusing on its relation to the development of cortical interneurons. This was achieved by in situ hybridization and immunohistochemistry in coronal cryosections of mouse telencephalon at early (E13.5), middle (E15.5) and late (E18.5) stages of embryonic cortical development.

**Fig. 1:**
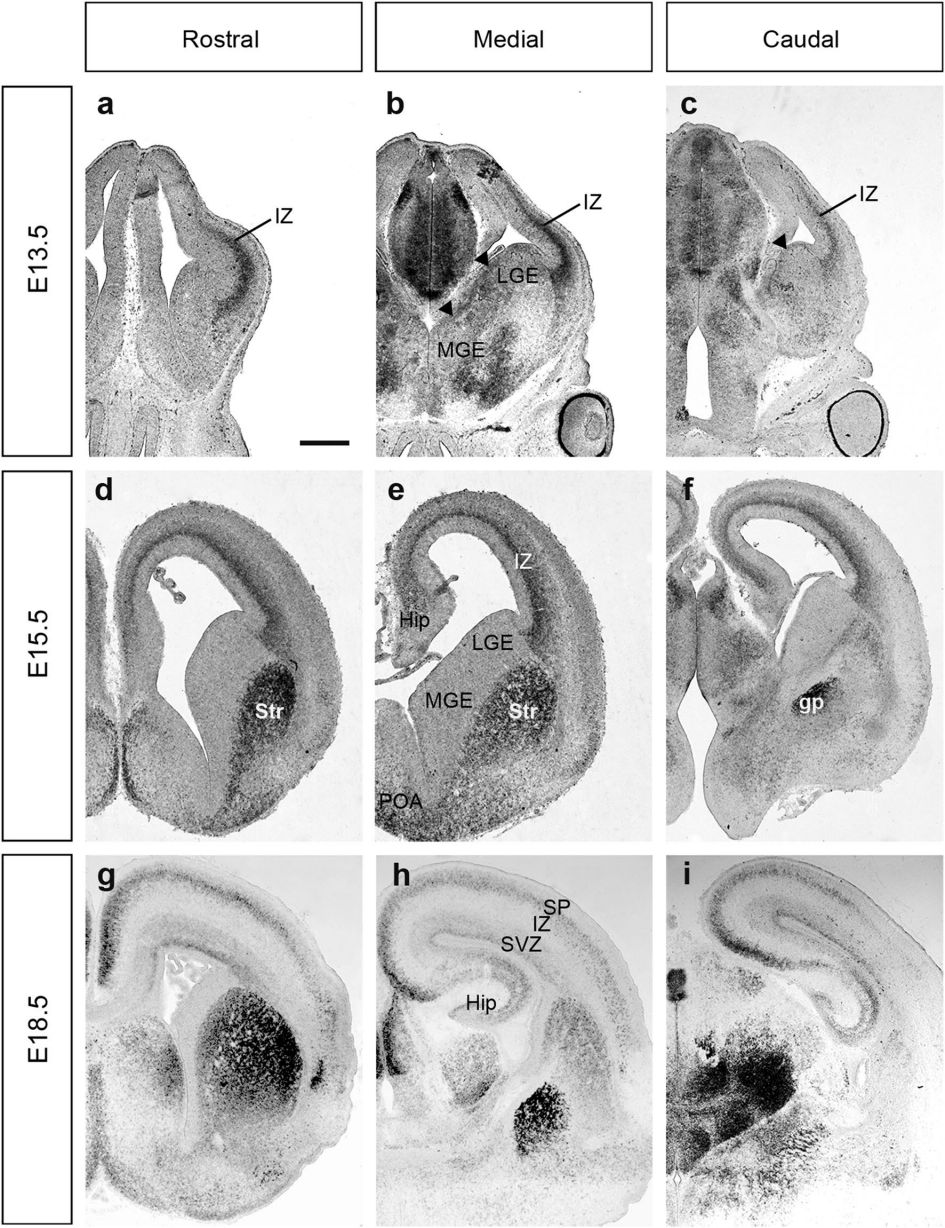
In situ hybridization for *Cdh8* mRNA expression in the developing forebrain. Coronal sections of the developing mouse forebrain at E13.5 (**a**–**c**), E15.5 (**d**–**f**) and E18.5 (**g**–**i**) showing the presence of *Cdh8* mRNA at rostral (**a, d, g**), medial (**b, e, h**) and caudal (**c, f, i**) levels. Arrowhead in b,c indicates expression in the proliferative zones. Scale bar in **a**: 500 µm and is applicable to all sections. *Gp* globus pallidus, *Hip* hippocampus, *IZ* intermediate zone, *LGE* lateral ganglionic eminence, *MGE* medial ganglionic eminence, *POA* preoptic area, *SP* subplate, *Str* striatum, *SVZ* subventricular zone

**Fig. 2:**
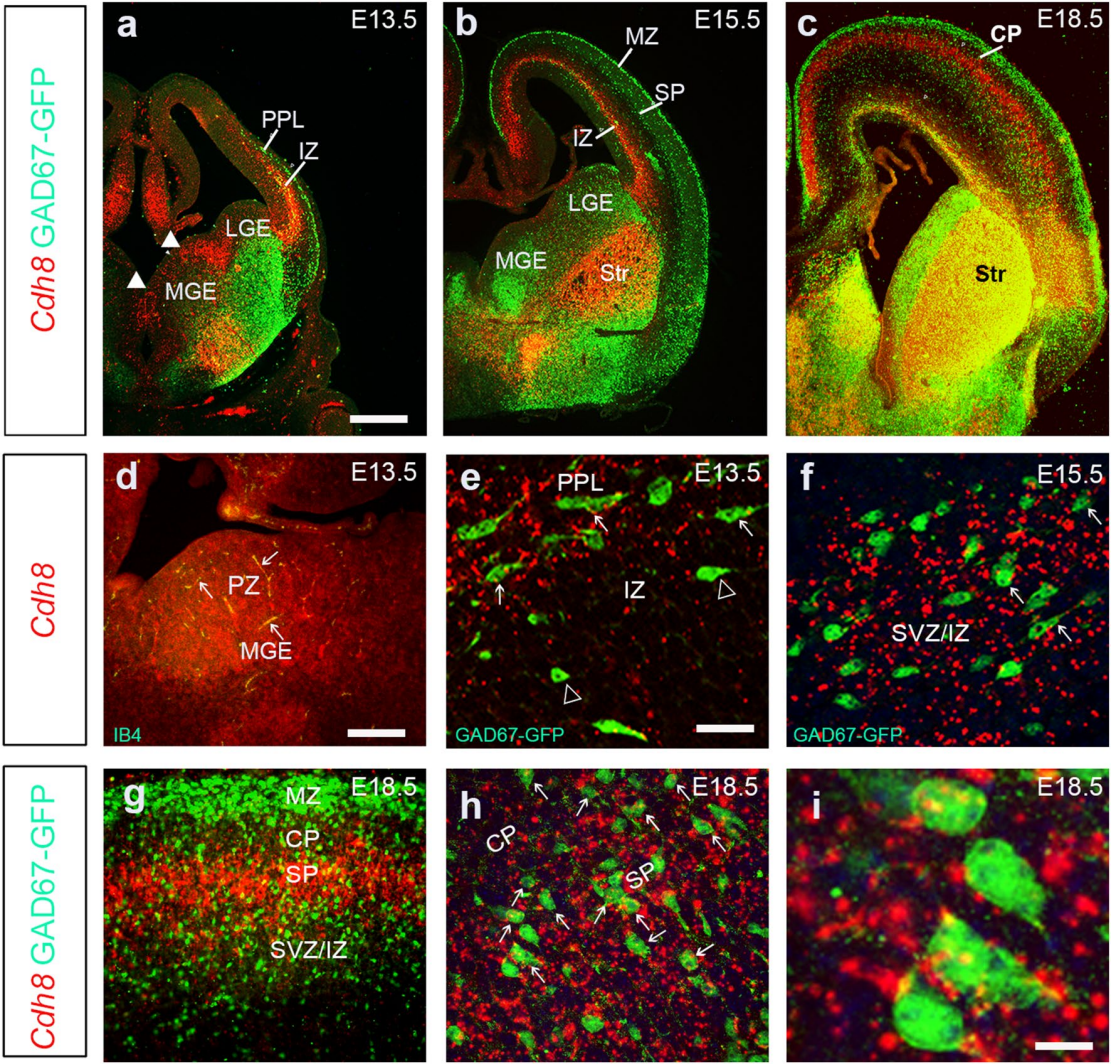
*Cdh8* is expressed at the site of origin of interneurons and along their migratory routes. Fluorescent in situ hybridization for *Cdh8* mRNA on coronal sections from GAD67-GFP mouse forebrains at E13.5 (**a, d, e**), E15.5 (**b, f**) and E18.5 (**c, g**–**i**), showing *Cdh8* expression (red) levels. Higher power images through cortex at E13.5 (**e**), E15.5 (**f**) and E18.5 (**g**–**i**); arrows indicate examples of interneurons that express *Cdh8* mRNA and hollow-arrowheads *Cdh8* negative interneurons. E13.5 Expression of *Cdh8* mRNA is observed in the proliferative zones (PZ), and to co-localize with the endothelial cell marker IB4 (**d**). Scale bar in **a** 500 µm (**a**–**c**); **d** is 200 µm (**d, g**); **e** is 100 µm (**e**–**h**), and **i** is 25 µm. *CP* cortical plate, *IZ* intermediate zone, *LGE* lateral ganglionic eminence, *MGE* medial ganglionic eminence, *MZ* marginal zone, *PPL* preplate layer, *Str* striatum, *SVZ* subventricular zone

At E13.5, there is a distinct pattern of *Cdh8* mRNA expression in cells of the IZ in rostral, medial and caudal cortical regions (Fig. 1a–c). This stripe of expression extends from the corticostriatal junction to approximately two-thirds of the length to the dorsal tip of the cortex, along a high ventral-low dorsal gradient. Interestingly, this approximates the distance cortical interneurons have migrated from the MGE at this age (Fig. 2a). In the ventral forebrain, *Cdh8* is localized in the subventricular and ventricular (SVZ and VZ) proliferative zones (PZ) of the ganglionic eminences (GE) (see arrowheads, Fig. 1b, c), suggesting that it may influence interneuron generation. In the medial and caudal sections, some weak staining in the mantles of the GEs can also be seen in agreement with previous work (Korematsu and Redies 1997a). Expression in the MGE interneuron progenitors was also confirmed by our recent RNA-sequencing data at E14.5 (unpublished data).

At E15.5, there is no longer any expression of *Cdh8* in the PZ of the MGE (Fig. 1d–f). Instead, the expression is now found in the preoptic area (POA) and strongly in the striatum in rostral and medial sections, and in the globus pallidus (gp) caudally. In the cortex, *Cdh8* is now more strongly expressed in the IZ, extending from the corticostriatal junction right around to the hippocampus throughout the entire rostro-caudal extent of the cortex (Fig. 1d–f). This, again, mirrors the distance that cortical interneurons have tangentially migrated in the IZ at this age (Fig. 2b).

By E18.5, *Cdh8* expression in the cortex has split from one defined streak in the IZ into two, which approximately correspond to the SVZ and the lateral IZ/SP (Fig. 1g–i). These are the two major interneuron migratory streams within the cortex before they move radially to populate the cortical plate (CP) (Faux et al. 2012). Thus, it appears that *Cdh8* mRNA is expressed in the germinal zones where interneurons are generated in early development, but also along their major migratory routes into the cortex, suggesting that this cadherin may play a role in interneuron generation as well as migration.

To investigate expression of *Cdh8* in interneurons themselves, we carried out fluorescent in situ hybridization in GAD67-GFP mice in which all interneurons express green fluorescent protein (Tamamaki et al. 2003). Our co-localization study at E13.5 shows that the expression of this cadherin closely follows the spatial distribution of cortical interneurons in the IZ stream (Fig. 2a). On closer examination, approximately 60% (58.28 ± 3.6%) of cells in the IZ appear to express *Cdh8* (arrows in Fig. 2e). In the ventral forebrain, we observe clear expression in the PZ and mantle of the MGE (white arrowheads in Fig. 2a), and colocalization with the endothelial cell marker IB4 (arrows in Fig. 2d). In the cortex at E15.5, *Cdh8* is expressed strongly in the IZ cortical interneuron migratory stream (Fig. 2b), with approximately 50% (50 ± 8.4%) of cells expressing *Cdh8* mRNA (arrows in Fig. 2f). By E18.5, individual streams of interneurons are difficult to discern, though approximately 77.6 ± 4.2% of GAD67-GFP^+^ cells in the CP/SP appear to express Cdh8 mRNA (Fig. 2c, g–i).

**Fig. 3:**
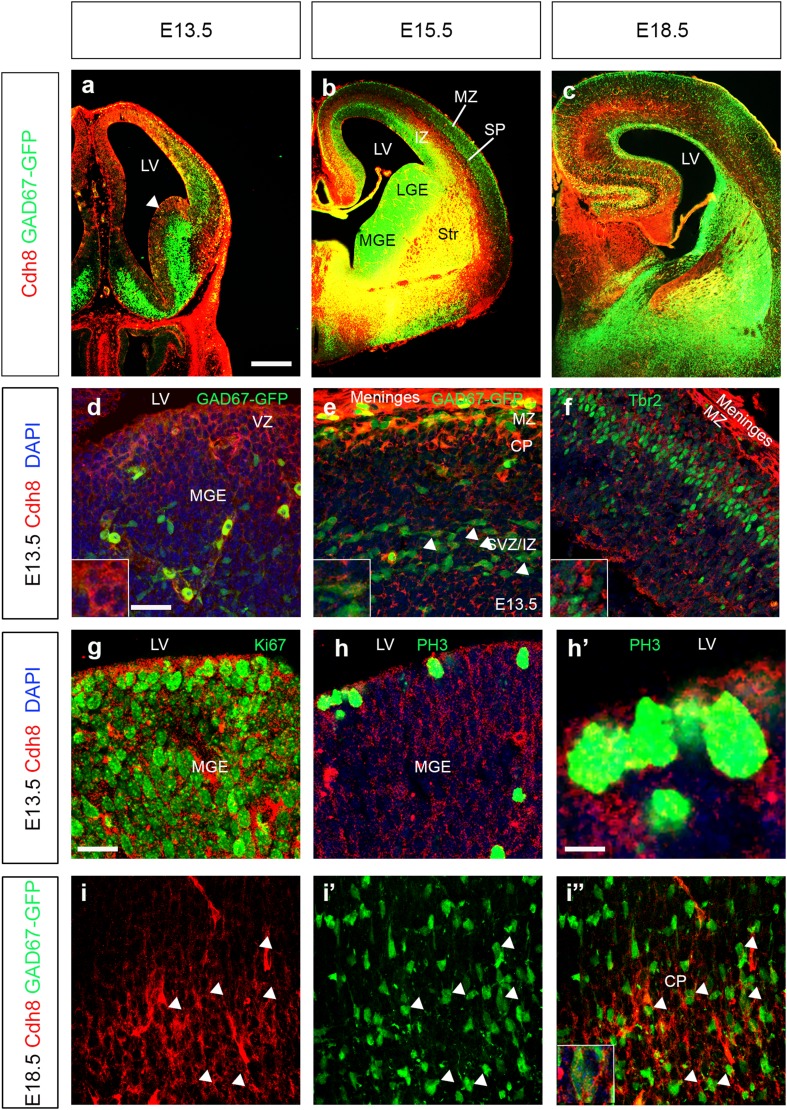
Expression of Cdh8 protein in cortical interneurons. Coronal sections from GAD67-GFP mouse forebrains at E13.5 (**a, d**–**h**′), E15.5 (**b**) and E18.5 (**c, i, i**″) were immunostained for Cdh8 protein (red). At E13.5, Cdh8 is strongly expressed in the ventral forebrain in the VZ of the MGE (**d**) and shows colocalization (yellow) with several proliferation markers Ki67 (**g**) and PH3 (**h, h**′). In the cortex, Cdh8 co-localizes in GAD67 positive cells in the SVZ/IZ layer (**e**), and with some Tbr2 expressing intermediate progenitors (**f**). At E15.5 (**b**) and E18.5 (**c**) co-localization is observed in the basal telencephalon. At E18.5, the majority of interneurons in the CP (arrowheads in **i, i**″) express Cdh8 protein. Scale bar in **a**, 500 µm (**a**–**c**), **d** is 75 µm (**d**–**f, i**,** i″**), g is 50 µm (**g, h**), **h**′ is 20 µm. *CP* cortical plate, *IZ* intermediate zone, *LGE* lateral ganglionic eminence, *LV* lateral ventricle, *MGE* medial ganglionic eminence, *MZ* marginal zone, *SP* subplate, *Str* striatum, *SVZ*/*IZ* subventricular/intermediate zone. *VZ* ventricular zone

The distribution of the Cdh8 protein mostly mirrors that of the mRNA throughout cortical development. This holds true particularly within the ventral forebrain, with strong expression seen in the MGE PZ at E13.5 (white arrowhead in Fig. 3a), and co-localization with several proliferation markers Ki67 and PH3 (Fig. 3g, h′) and within the POA and striatum at E15.5 and E18.5 (Fig. 3b, c). However, it does differ in certain aspects, for example, whilst in situ hybridization at E13.5 and E15.5 shows expression in an area tightly restricted to the IZ stream, the antibody staining is much broader medio-laterally, perhaps along axonal projections within the cortex (Fig. 3e), and in some intermediate Tbr2 expressing progenitors (Fig. 3f). Approximately 70% (66.67 ± 4.85%) of interneurons in the SVZ/IZ appear to express Cdh8 at E13.5 (white arrowheads in Fig. 3e), in a punctate manner, similar to cultured primary pyramidal neurons as previously reported (Friedman et al. 2015). The broader expression pattern of Cdh8 in the ventral forebrain appears to overlap with GAD67-GFP, at E15.5 and E18.5, suggesting that many migrating interneurons express this cadherin. Indeed, at E18.5, the majority of interneurons in the CP do appear to express Cdh8 (77.78 ± 9.2%) protein (white arrowheads in Fig. 3i, i″).

### Altered interneuron number in the cortex of *Cdh8*^−/−^ mice

**Fig. 4:**
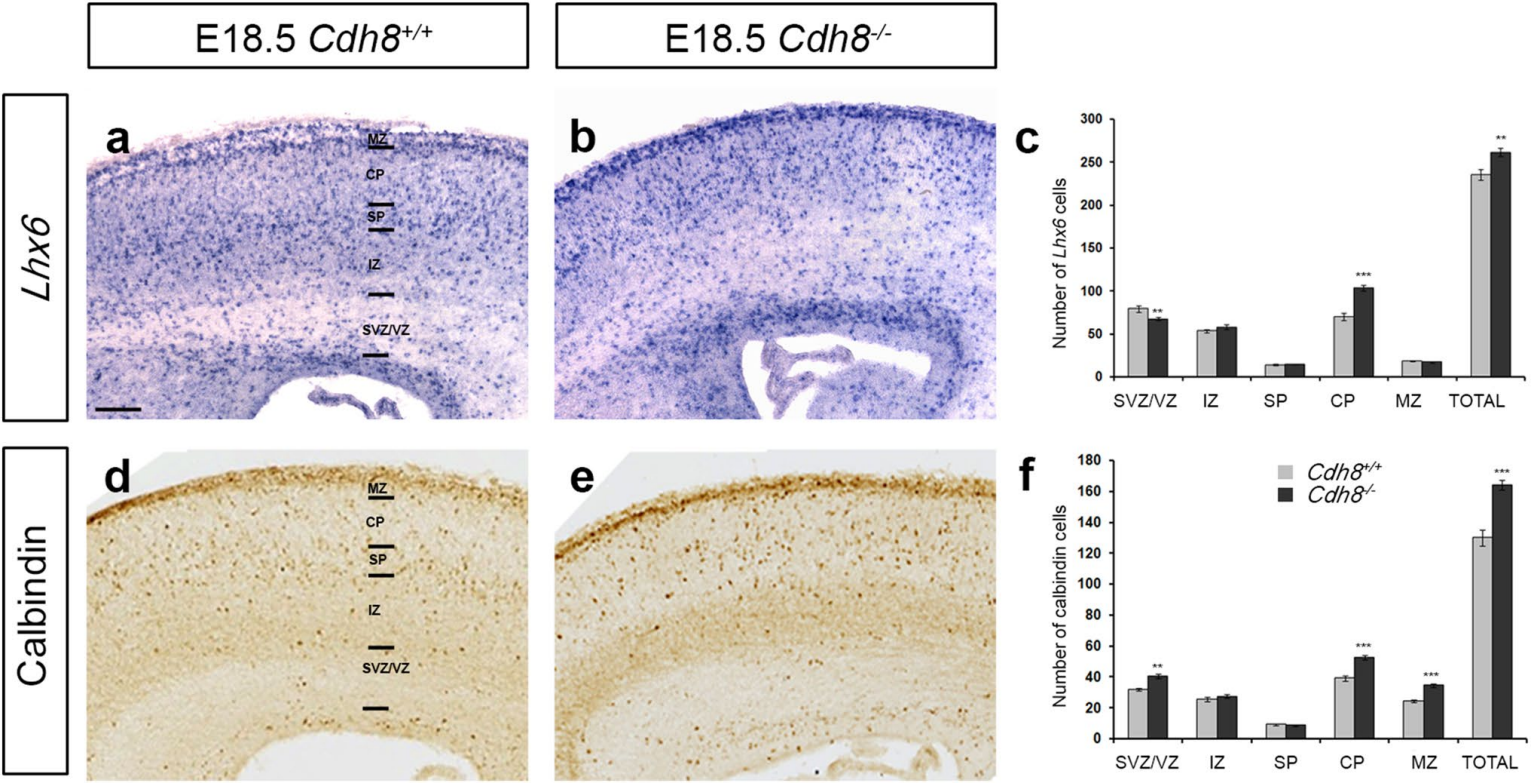
Increased number of interneurons in the cortex of *Cdh8*^−/−^ mice. Images of in situ hybridization for *Lhx6* (**a, b**) and immunohistochemistry for calbindin (**d, e**) in **c**oronal brain sections from *Cdh8*^+/+^ (**a, d**) and *Cdh8*^−/−^ (**b, e**) mice at E18.5. **c, f** Quantification of *Lhx6* (**c**) and calbindin (**f**) positive cells in the cortex show an increased number at E18.5 when compared to *Cdh8*^+/+^ controls. Scale bars in **a** is 200 µm (**p < 0.001, ***p < 0.0001). Error bars indicate SEM. *CP* cortical plate, *IZ* intermediate zone, *MZ* marginal zone, *SVZ*/*VZ* subventricular zone/ventricular zone

To determine if Cdh8 has an effect on cortical development, we first looked at the number and position of interneurons in the developing cortex of *Cdh8*^+/+^ and *Cdh8*^−/−^ mice, at late (E18.5) stage of development (*n* = 3 for each genotype), when we see the strongest expression of this gene in these cell types. Using in situ hybridization and immunochemistry for interneuron markers *Lhx6* (Alifragis et al. 2004) and calbindin (CB) (Anderson et al. 1997), we assessed the number of interneurons within the developing cortex. We observed a significant increase in the total number of both *Lhx6*^+^ (*Cdh8*^+/+^ 235.84 ± 6.23; *Cdh8*^−/−^ 261.5 ± 4.89, *p* < 0.0024) and CB^+^ (*Cdh8*^+/+^ 130.18 ± 5.17; *Cdh8*^−/−^164.1 ± 3.04, *p* < 0.0004) and cells in the cortex at E18.5 (Fig. 4a–f). The distribution of *Lhx6* cells within the different layers of the developing cortex was also altered, with a significant increase in cells in the CP (*Cdh8*^+/+^ 70.2 ± 3.95; *Cdh8*^−/−^103.65 ± 3.22, *p* < 0.00005) (Fig. 4c). We also observed changes in the distribution of CB^+^ cells within cortical layers, with a significant increase in cells in the SVZ/IZ (*Cdh8*^+/+^ 31.82 ± 1.01; *Cdh8*^−/−^ 40.5 ± 1.38, *p* < 0.009), CP (*Cdh8*^+/+^ 39.27 ± 1.71; *Cdh8*^−/−^ 52.7 ± 1.27, *p* < 0.0006) and marginal zone (MZ) (*Cdh8*^+/+^ 24.36 ± 0.86; *Cdh8*^−/−^ 34.6 ± 1.28, *p* < 0.0006) (Fig. 4f).

The increase in the number of *Lhx6*^+^ and CB^+^ cells migrating into the neocortex of *Cdh8*^−/−^ mice could be due to an increase in proliferation or reduction in cell death. We suggest that the latter possibility is rather unlikely in view of the fact that, overall, little apoptosis is observed in the developing cortex, apart from the SVZ at birth and in the first two postnatal weeks (Thomaidou et al. 1997; Southwell et al. 2012). Increased proliferation is the most probable cause, as Cdh8 is expressed in the PZ of the GE at early stages of corticogenesis, suggesting it could regulate the proliferation of interneuron progenitors.

**Fig. 5:**
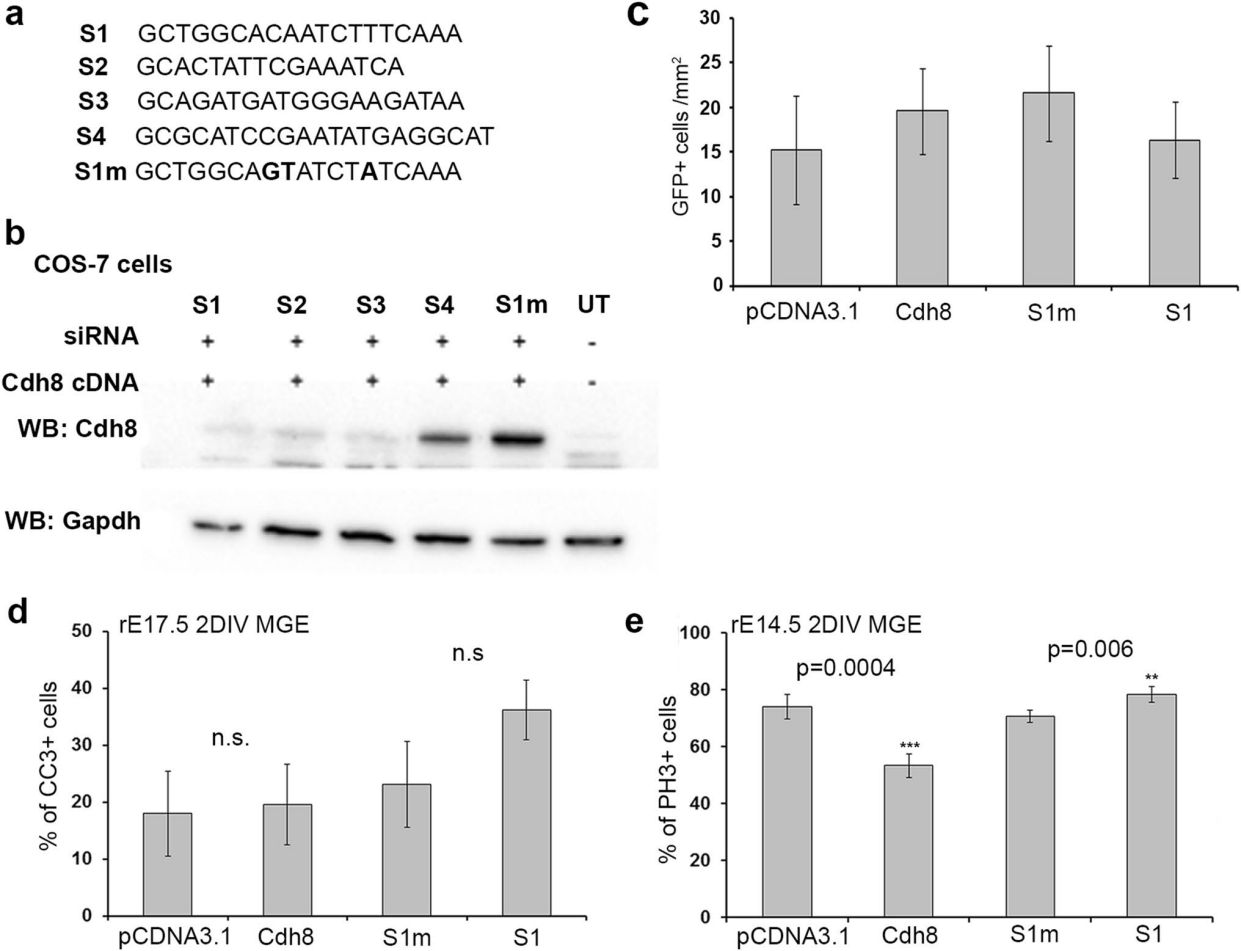
Suppression of Cdh8 protein expression by siRNAs affects the proliferation of interneuron progenitors. **a** Sequences of specific and triple-point-mutated siRNAs targeting mouse Cdh8 mRNA. **b** Western blot shows the effectiveness of S1, S2 and S3, but not S4 or S1-mutated (S1m) sequence at knocking down Cdh8 mRNA expression in COS-7 cells. **c** Quantification of rE17.5 MGE interneuron cell migration in a Boyden’s chamber following transfection with either Cdh8 over expressing, knockdown or control constructs. **d, e** Histogram shows the percentage of rE17.5 MGE interneuron cultures (CB^+^/GFP^+^) that are apoptotic (CC3^+^) (**d**), and rE14.5 MGE neuronal progenitors (Nestin^+^/GFP^+^) that are proliferating (PH3^+^) (**e**), following 2 days in vitro (2DIV) after transfection with either over-expressing, knockdown or control constructs (**p < 0.001, ***p < 0.0001). Error bars indicate SEM

As we were unable to obtain additional knockout tissue for further experiments, we decided to apply the RNAi technique to investigate the role(s) of Cdh8 in corticogenesis. We designed four RNAi sequences (called S1–S4) that target four different regions within *Cdh8* mRNA (Fig. 5a). To test the effectiveness of our siRNA constructs on Cdh8 expression, we transfected COS-7 cells with the over expressing, knockdown and mutated siRNA constructs (at a ratio of 1:3). 48 h after transfection, we assessed the level of Cdh8 mRNA and protein in these cells. We observed a significant reduction in both Cdh8 mRNA and protein with knockdown constructs S1–S3, but not S4 or with mutated S1 sequence (S1m), which can serve as control for the siRNA transfections (Fig. 5b). These constructs were used in further in vitro experiments to assess the role of Cdh8 in proliferation and apoptosis.

### Loss of Cdh8 function increases proliferation in neuronal cultures

To examine the effects of over expressing or silencing Cdh8 on cell migration, proliferation and apoptosis, Cdh8-GFP cDNA or control (pCDNA3.1-GFP), and S1 siRNA (GFP) or mutated Sl siRNA (GFP) (S1m) were introduced into E14 rat (equivalent to mouse E12.5) MGE-dissociated neural progenitors in order to examine proliferation or into E17 (equivalent to mouse E15.5) MGE cultures to assess for migration and apoptosis. After 2 days in vitro (2DIV), cells were stained for Nestin (neuronal progenitor marker) and PH3 (proliferation marker) to look at proliferation, or CB (interneuron) and CC3 (apoptotic) markers to identify apoptotic interneurons. Over-expressing (pCDNA3.1 18.01 ± 7.43%, Cdh8 19.64 ± 7.07%, *p* < 0.2) or knocking down (S1m 23.15 ± 7.5%, S1 36.21 ± 5.21%, *p* < 0.165) Cdh8 expression did not have a significant effect on the level of apoptosis in our cultures (Fig. 5d). However, altering Cdh8 levels had a significant effect on proliferation. Over expressing Cdh8 resulted in decreased (pCDNA3.1 73.98 ± 4.24%, Cdh8 53.3 ± 4.12%, *p* = 0.0004), whilst knockdown led to increased proliferation (S1m 70.64 ± 2.25%, S1 78.23 ± 2.8%, *p* = 0.006) of the transfected progenitors in the dissociated cultures (Fig. 5e). To assess migration we used a chemomigration assay. In these experiments we found alteration of Cdh8 levels in MGE interneurons had no affect on their migratory potential (Fig. 5c) These findings are in agreement with our knockout mouse studies, where we observe increased number of interneurons in the cortex, most probably due to increased proliferation and generation of more interneurons.

**Fig. 6:**
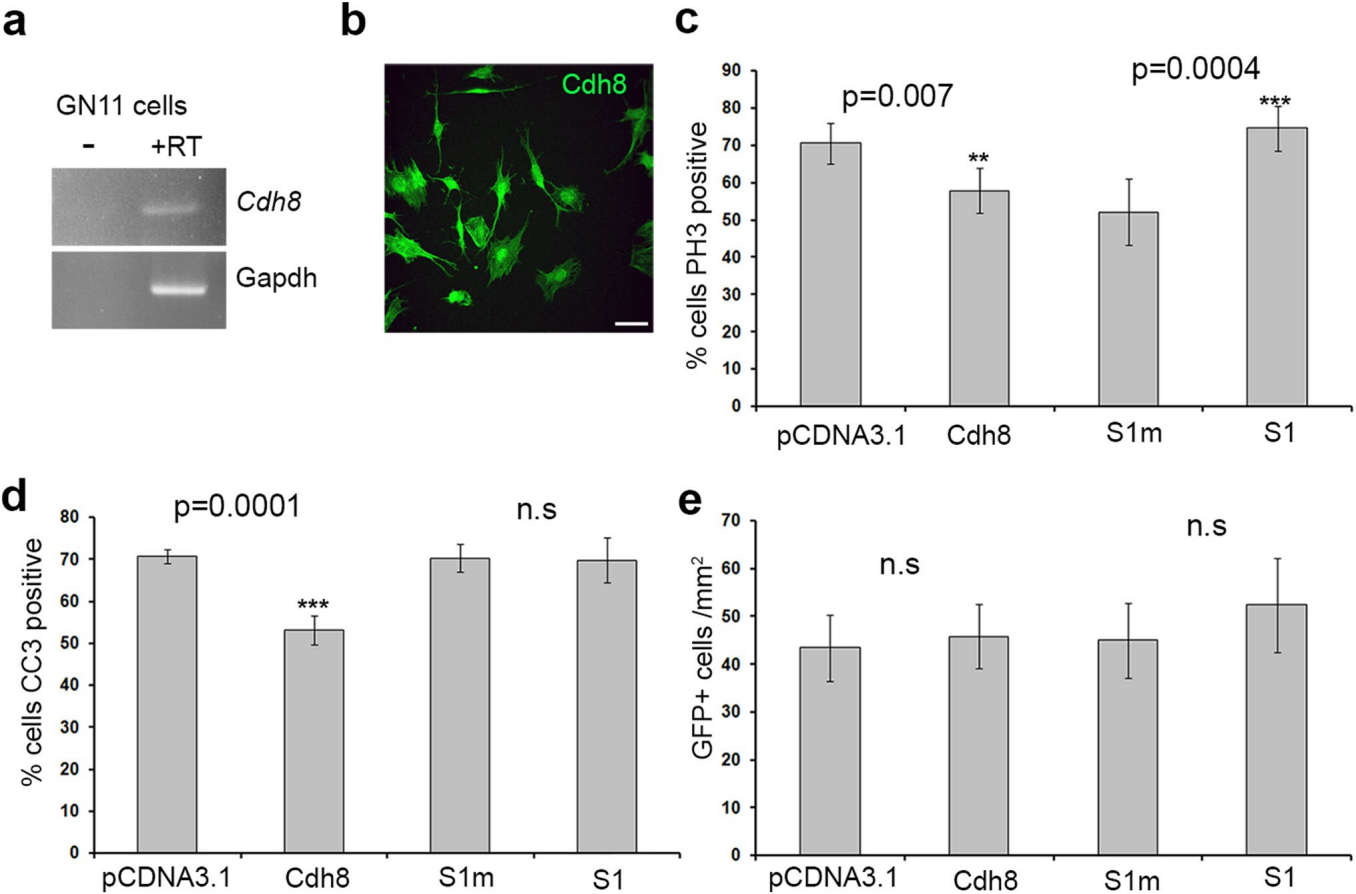
Cdh8 is expressed by GN11 cells and affects their proliferation. **a** Reverse transcription-PCR analysis showed expression of *Cdh8* in GN11 cells. **b** Immunohistochemistry confirmed the expression of Cdh8 in GN11 cells. **c, d** Histograms show percentage of proliferating (GFP^+^/PH3^+^) (**c**), and apoptotic (GFP^+^/CC3^+^) (**d**) cells following transfection with either over expressing, knockdown or control constructs. **e** Quantification of GN11 cell migration in a Boyden’s chamber following transfection with either Cdh8 over expressing, knockdown or control constructs. Scale bar in *b* is 100 µm. (**p < 0.001, ***p < 0.0001). Error bars indicate SEM

Next, we wanted to determine whether the observed increase in proliferation was specific to cortical interneurons or whether it applied to other migrating neuronal cell types. In previous studies, we have used the immortalized gonadotropin-releasing hormone secreting (GnRH) neurons (GN11 cells) to look at the effects of factors on migration (Hernandez-Miranda et al. 2011) and on proliferation/apoptosis (Cariboni et al. 2011), as they share a common repertoire of genes with cortical interneurons and exhibit a similar migratory behavior. We found that these cells, such as interneurons, expressed Cdh8 mRNA as established by reverse transcription-PCR (Fig. 6a) and protein by immunohistochemistry (Fig. 6b).

In terms of proliferation we observed a small, but significant decrease in the number of PH3^+^ cells following over-expression of Cdh8 (pCDNA3.1 70.57 ± 5.44%, Cdh8 57.8 ± 5.99%, *p* = 0.007) in GN11 cells (Fig. 6c). Interestingly, Cdh8 knockdown resulted in increased proliferation (S1m 52.14 ± 8.85%, S1 74.59 ± 5.98%, *p* = 0.0004) of the transfected cells in the GN11 cultures, which is in agreement with our observations in MGE neuronal progenitors. The effect of altering Cdh8 levels on GN11 cell proliferation appears stronger than on interneuron progenitors; possibly due to the homogeneity of the GN11 population (all cells express Cdh8). In our hypoxic cultures, apoptosis was reduced when Cdh8 was over-expressed (CC3: pCDNA3.1 70.78 ± 1.68%, Cdh8 53.15 ± 3.35%, *p* = 0.0001), but not when it was knocked down (S1m 70.22 ± 3.28%, S1 68.76 ± 5.32%, *p* = 0.89, Fig. 6d).

A previous study demonstrated that over-expression of two dominant negative constructs of NCAD and CAD11 slowed down the migration of lateral reticular nucleus and external cuneate nucleus (LRN/ECN) neurons, while over-expression of these cadherins had no effect (Taniguchi et al. 2006). Here, we wanted to see if altering Cdh8 levels affected GN11 cell migration using a chemomigration assay. We found that altering Cdh8 expression in these cells had no effect on their migratory ability (pCDNA3.1 43.45 ± 6.97%, Cdh8 45.83 ± 6.7%, *p* = 0.808; S1m 45.04 ± 7.85%, S1 52.38 ± 9.88%, *p* = 0.57) (Fig. 6e), supporting the idea that Cadh8 is not involved in neuronal migration, which is in agreement with our interneuron migration data (Fig. 5c). Taken together, our data suggest that altering Cdh8 levels significantly affects their proliferative potential.

**Fig. 7:**
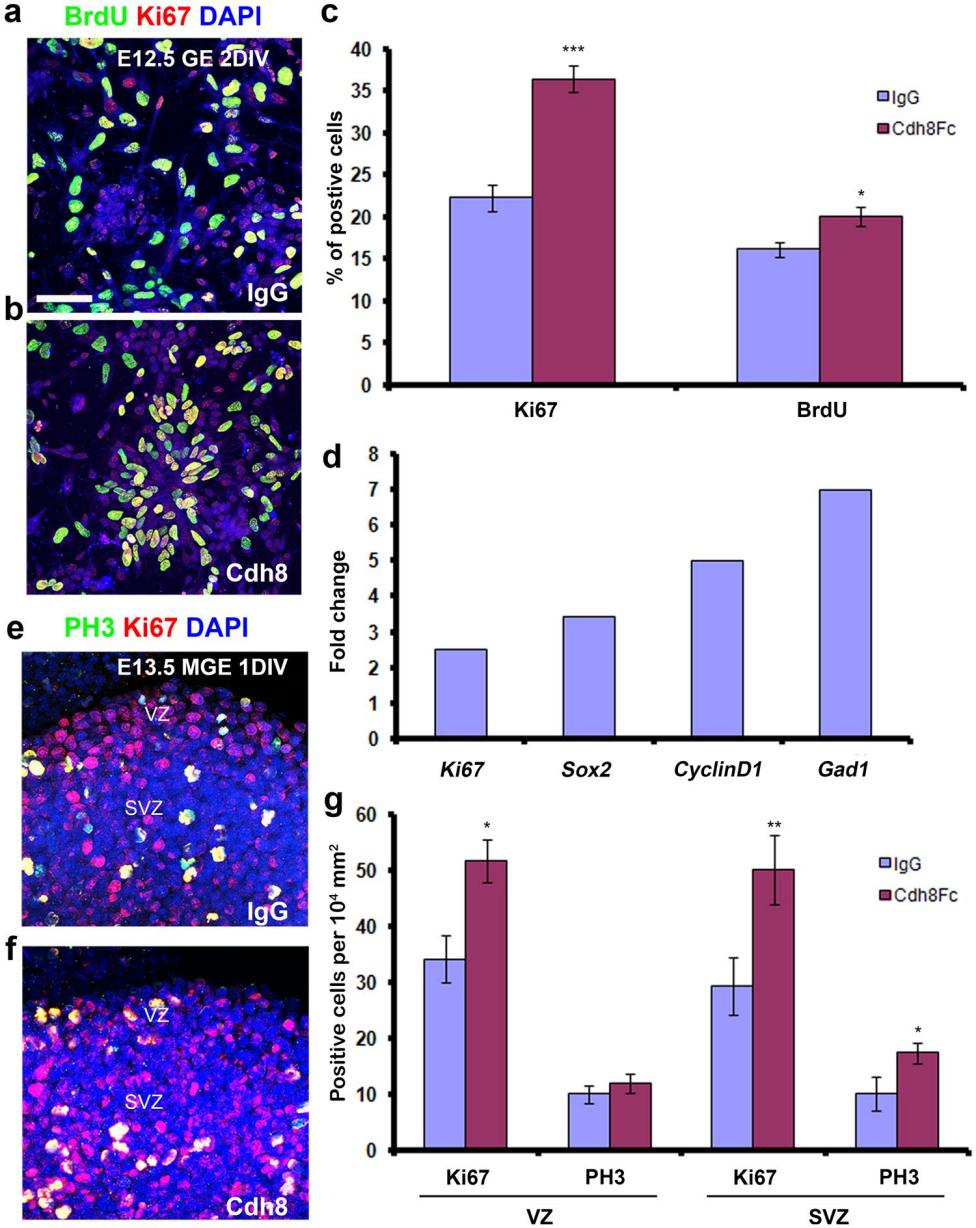
Proliferation of cortical interneuron progenitors is increased after treatment with Cdh8-Fc. **a, b** E12.5 mouse GE-derived dissociated interneuron progenitor cultures treated with IgG (control) or Cdh8-Fc were labelled for BrdU and Ki67. **c** Graph shows increased numbers of BrdU^+^ and Ki67^+^ proliferating cells following Cdh8-Fc treatment. **d** Quantitative-PCR shows increased levels of expression for proliferation, cell cycle, stem cell and interneuron genes, in cells treated with Cdh8-Fc for 2DIV when compared to treatment with IgG (control). **e, f** E13.5 mouse cortical slices were treated for 24 h with IgG (**e**) or Cdh8-Fc (**f**) were labelled with anti-PH3 or Ki67 antibody, photographed at the level of MGE. **g** Quantification of Ki67^+^ and PH3^+^ cells in the VZ and SVZ of MGE revealed a significant increase in the proliferation of MGE cells after Cdh8-Fc treatment. Scale bar in **a**, 50 µm (**a, b, e, f**) (*p < 0.01, **p < 0.001, ***p < 0.0001). Error bars indicate SEM. *SVZ* subventricular zone, *VZ* ventricular zone

To confirm these results, we used an alternative approach that does not rely on the transfection efficiency of the dissociated progenitors, but in the functional blocking of Cdh8 through the application of Cdh8-Fc protein. Interneuron progenitors dissociated from mouse E12.5 GEs were treated with 1 µg/ml Cdh8-Fc or IgG (control) for two DIV and BrdU was added to the media 2 h before fixation. Counts of cells stained for BrdU or the general proliferation marker Ki67 showed increased proliferation in Cadh8-Fc treated cultures when compared to control treatments (Ki67: Cdh8-Fc 36.45 ± 1.42%, IgG 22.3 ± 1.6%, *p* = 0.0001, BrdU: Cdh8-Fc 20.1 ± 1.2%, IgG 16.2 ± 0.9%, *p* = 0.02, *n* = 3) (Fig. 7a–c). These results were further corroborated by qPCR with RNA extracted from Cdh8-Fc treated cultures versus IgG controls. Genes involved in proliferation, cell cycle progression and pluripotency showed an increase in their expression (Ki67 twofold, Sox2 threefold, CyclinD1 fivefold, Fig. 7d); Gad1 also showed a sevenfold increase in expression, suggesting an increase in the number of interneurons in these cultures (Fig. 7d). Cell cycle kinetics analysis showed that the cycle length was not affected in the Cdh8-Fc treated cells (when compared to controls), but fewer cells exited the cycle in the Cdh8-Fc treated cultures (Cdh8-Fc 6.1%, IgG 11.5%). Finally, to further verify the effect of Cdh8 on the proliferation of interneurons, we treated E13.5 mouse cortical slices with Cdh8-Fc or IgG for 24 h and counted the number of proliferating cells in the VZ and SVZ in both conditions. We found that Cdh8-Fc treated slices had increased numbers of Ki67^+^ cells in the VZ and SVZ of the MGE (Ki67, VZ: Cdh8-Fc 51.6 ± 3.8, IgG 34.2 ± 4.2, *p* = 0.02, SVZ: Cdh8-Fc 50.1 ± 6.1, IgG 29.3 ± 5.2, *p* = 0.0012) and more PH3^+^ cells in the SVZ (PH3^+^: Cdh8-Fc 18.33 ± 1.8, IgG 10.8 ± 1.99, *p* = 0.01) (Fig. 7e–g).

Taken together, our data suggest that Cdh8 does not play a significant role in migration or apoptosis of interneurons within the developing cortex or in vitro, but altering Cdh8 levels significantly affects the proliferative potential of these cells.

## Discussion

Corticogenesis involves the production, maintenance and arrangements of pyramidal neurons and interneurons into precise cortical circuits. Disruption to this delicate balance may result in wide-ranging neurological and cognitive defects. Thus, there has been considerable interest over the years on the molecular mechanisms that underlie their tangential and radial migration, and an ever-increasing list of molecules that play important roles in their generation, differentiation and migration (Faux et al. 2012; Marin 2013; Guo and Anton 2014). However, little is known about the molecules involved in interneuron migratory stream specification. In an attempt to address this point, we previously carried out a microarray study and identified several members of the cadherin family which were differentially expressed, including Cdh8.

Cadherins are a superfamily of more than 100 transmembrane glycoproteins originally identified as cell adhesion molecules (Takeichi 2007; Hirano and Takeichi 2012). Functional studies have revealed that they are involved in neural patterning, cell migration, axon guidance, and synapse formation and function (Nakagawa and Takeichi 1998; Manabe et al. 2000; Inoue et al. 2001; Treubert-Zimmermann et al. 2002; Suzuki et al. 2007; Barnes et al. 2010; Osterhout et al. 2011; Williams et al. 2011; Kuwako et al. 2014). With the exception of a few cadherins, such as N-cadherin (Luccardini et al. 2013) and Celsr3 (Ying et al. 2009), which have been shown to regulate cortical interneuron migration in the developing mouse brain, not much is known about how this class of molecules affect the development and/or migration of these neurons.

Cdh8 merited further study as it has recently been implicated in the susceptibility to autism and learning difficulties (Pagnamenta et al. 2011), disorders that may be attributed to defects in interneuron development. A plethora of mutations have been observed in the T-Brain (*TBR1*) gene in patients with autism spectrum disorders (Neale et al. 2012; O’Roak et al. 2012a, b), and *Tbr1*^+/−^ mice serve as a model for autism such as behaviours (Huang et al. 2014). More recently, downstream targets of Tbr1 have been identified using a microarray and promoter analysis in Tbr1 deficient mice, and one of the genes identified was *Cdh8* (Chuang et al. 2015). A recent knockdown study implicated *Cdh8* in the regulation and generation of normal cortical projections, neuronal morphology and cortico-striatal synapses (Friedman et al. 2015), which are features affected in autism patients, suggesting that Cdh8 may play a role in corticogenesis and in the development of the disorder. However, the role of Cdh8 in interneuron development and potential contribution to autism has not been assessed.

To address this, we first used in situ hybridization and immunohistochemistry to expand on previous studies (Korematsu and Redies 1997a, b; Korematsu et al. 1998a, b; Medina et al. 2004; Lefkovics et al. 2012) and examined the expression of Cdh8 during cortical development. These experiments revealed the presence of Cdh8 in progenitor cells lining the ventricular surface of the GEs as early as E13.5, as well in the majority of migrating interneurons, suggesting it may play a role in their generation and/or migration.

To assess whether Cdh8 affect interneuron development, we analyzed mice depleted of this cadherin. This analysis of *Cdh8*^−/−^ mice revealed an increased number of interneurons in the cortex at the end of corticogenesis, a defect that could be due to a number of factors including altered migration, reduced apoptosis and/or increased proliferation. Cdh8 has been shown to play a role in the migration of facial branchiomotor neurons in the mouse hindbrain (Garel et al. 2000) and, more recently, has been implicated in the migration of facial neurons, as volproic acid treatment hindered their migration, which coincided with decreased *Cdh8* mRNA levels (Oyabu et al. 2013). Cdh8 has also been implicated in metastasis in non-small cell lung cancer (Lu et al. 2006). Whilst these studies have suggested a role for Cdh8 in cell migration, our in vitro experiments did not support such a role in cortical interneurons or GN11 cells, indicating either altered apoptosis or proliferation are most likely responsible for the increased number of interneurons observed in the cortex of *Cdh8*^−/−^ mice. Since apoptosis in developing cortical interneurons occurs mainly in the first postnatal days and is intrinsically regulated (Southwell et al. 2012; Thomaidou et al. 1997), we reasoned that the increased number of interneurons in the E18.5 *Cdh8*^−/−^ mice is not due to reduced apoptosis. Indeed, reduction of Cdh8 levels in *our vitro* experiments did not affect interneuron apoptosis.

The expression of Cdh8 in the proliferative zones of the MGE points to a role in proliferation. Indeed, we observed in our in vitro experiments that over the expression of Cdh8 levels decreased proliferation and knockdown had the opposite effect. This latter finding is in accordance with recent studies which demonstrated that knocking down CDH8 expression in gastric cancer cell lines, MKN45 and NUGC4, promoted proliferation (Sawada et al. 2013). Similarly, expression of a dominant negative form of Ncad in chicken spinal cord caused loss of cadherin-based adhesions in the apical end-feet of neuron progenitors, which resulted in increased proliferation and neuron numbers (Hatakeyama et al. 2014). Furthermore, removal of Cdh2 leads to dramatic increase in proliferation of cortical neurons, leading to the formation of a double-cortex (Gil-Sanz et al. 2014), highlighting the importance of maintenance of adherens junctions for the control of cell proliferation in the developing brain. On the other hand, Cadh13 was implicated in the regulation of programmed cell death and survival in the developing forebrain (Killen et al. 2017), pointing to the many different or even opposite functions of Cadherins during brain development. In summary, we report here that Cdh8 deficient mice have increased numbers of interneurons in their cortex due to increased proliferation of their progenitors. Our findings support a novel role for Cdh8 in neurogenesis in the ventral telencephalon during development.
